# 2,2-Dibenzyl­hydrazin-1-ium chloride

**DOI:** 10.1107/S1600536813003966

**Published:** 2013-02-16

**Authors:** Shahedeh Tayamon, Thahira Begum S. A. Ravoof, Mohamed Ibrahim Mohamed Tahir, Karen A. Crouse, Edward R. T. Tiekink

**Affiliations:** a Department of Chemistry, Universiti Putra Malaysia, 43400 Serdang, Malaysia; b Department of Chemistry, University of Malaya, 50603 Kuala Lumpur, Malaysia

## Abstract

In the title salt, C_14_H_17_N_2_^+^·Cl^−^, the central N atom is pyramidal (sum of bond angles = 330.9°) and there is a near orthogonal relationship between the benzene rings [dihedral angle = 89.95 (10)°]. The crystal packing features N—H⋯Cl hydrogen bonds, which lead to a supra­molecular undulating ribbon along the *a* axis comprising edge-shared eight-membered {⋯HNH⋯Cl}_2_ synthons. The chains are connected into layers in the *ab* plane by C—H⋯π inter­actions.

## Related literature

For background to the synthesis of *S*-substituted dithio­carbaza­tes and their metal complexes, see: Ravoof *et al.* (2010 ▶); Tayamon *et al.* (2012 ▶). For the synthesis, see: Tarafder *et al.* (2000 ▶). For the structure of the diphenyl analogue of the cation, see: Stender *et al.* (2003 ▶).
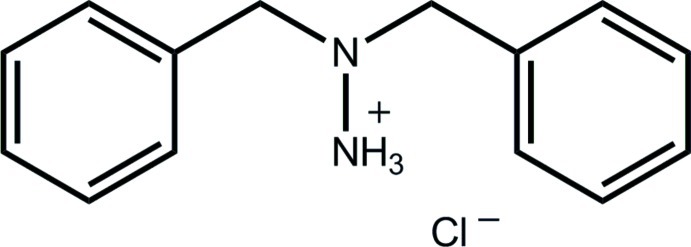


## Experimental

### 

#### Crystal data

C_14_H_17_N_2_^+^·Cl^−^
*M* *_r_* = 248.75
Triclinic, 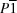

*a* = 5.6155 (4) Å
*b* = 9.9804 (7) Å
*c* = 11.7302 (9) Å
α = 79.532 (6)°
β = 78.508 (6)°
γ = 83.550 (6)°
*V* = 631.54 (8) Å^3^
*Z* = 2
Cu *K*α radiation
μ = 2.49 mm^−1^
*T* = 100 K
0.14 × 0.09 × 0.02 mm

#### Data collection

Oxford Diffraction Xcalibur Eos Gemini diffractometer
Absorption correction: multi-scan (*CrysAlis PRO*; Agilent, 2011 ▶) *T*_min_ = 0.72, *T*_max_ = 0.95
6961 measured reflections
2407 independent reflections
2076 reflections with *I* > 2σ(*I*)
*R*_int_ = 0.034

#### Refinement

*R*[*F* ^2^ > 2σ(*F* ^2^)] = 0.047
*wR*(*F* ^2^) = 0.129
*S* = 1.07
2407 reflections
166 parameters
H atoms treated by a mixture of independent and constrained refinement
Δρ_max_ = 0.45 e Å^−3^
Δρ_min_ = −0.24 e Å^−3^

### 

Data collection: *CrysAlis PRO* (Agilent, 2011 ▶); cell refinement: *CrysAlis PRO*; data reduction: *CrysAlis PRO*; program(s) used to solve structure: *SHELXS97* (Sheldrick, 2008 ▶); program(s) used to refine structure: *SHELXL97* (Sheldrick, 2008 ▶); molecular graphics: *ORTEP-3 for Windows* (Farrugia, 2012 ▶) and *DIAMOND* (Brandenburg, 2006 ▶); software used to prepare material for publication: *publCIF* (Westrip, 2010 ▶).

## Supplementary Material

Crystal structure: contains datablock(s) global, I. DOI: 10.1107/S1600536813003966/hb7036sup1.cif

Structure factors: contains datablock(s) I. DOI: 10.1107/S1600536813003966/hb7036Isup2.hkl

Supplementary material file. DOI: 10.1107/S1600536813003966/hb7036Isup3.cml

Additional supplementary materials:  crystallographic information; 3D view; checkCIF report

## Figures and Tables

**Table 1:** Hydrogen-bond geometry (Å, °) *Cg*1 is the centroid of the C9–C14 phenyl ring.

*D*—H⋯*A*	*D*—H	H⋯*A*	*D*⋯*A*	*D*—H⋯*A*
N2—H1N⋯Cl1	0.94 (3)	2.30 (3)	3.2130 (18)	163 (2)
N2—H2N⋯Cl1^i^	0.97 (2)	2.21 (2)	3.1287 (19)	158 (2)
N2—H3N⋯Cl1^ii^	0.93 (3)	2.20 (3)	3.1235 (18)	172 (2)
C8—H8*A*⋯*Cg*1^iii^	0.99	2.64	3.542 (2)	152

Symmetry codes: (i) 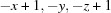; (ii) 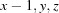; (iii) 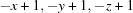.

**Fig. 1.**
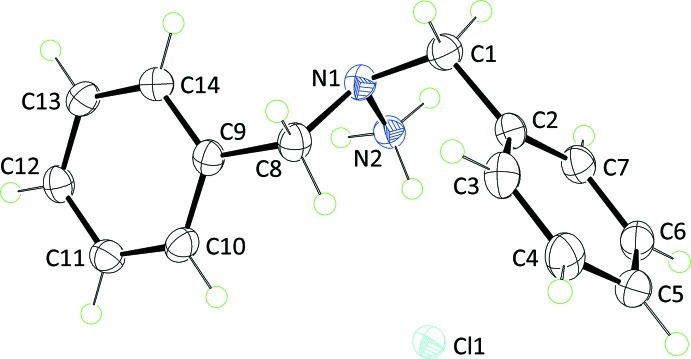
The molecular structure of salt (I) showing displacement ellipsoids at the 50% probability level.

**Fig. 2.**
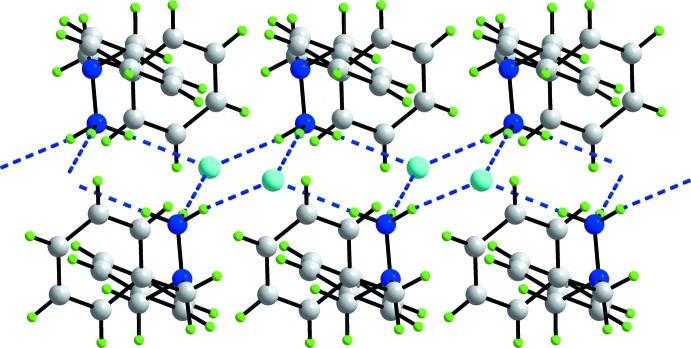
A view of the undulating supramolecular chain in (I) mediated by N—H···Cl hydrogen bonds (blue dashed lines) along the a axis.

**Fig. 3.**
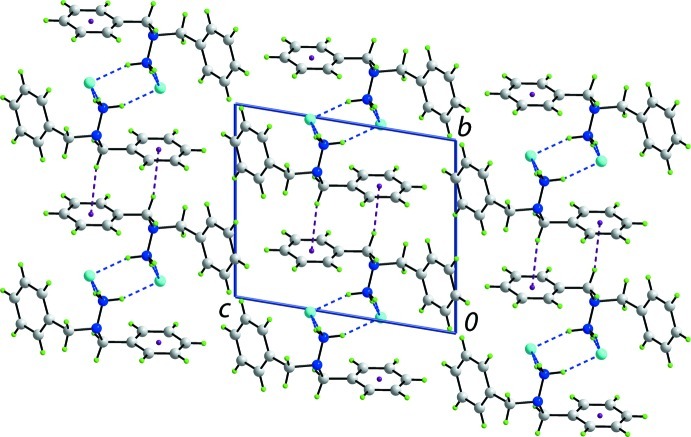
A view of the crystal packing in projection down the a axis. The N—H···Cl and C—H···π interactions are shown as blue and purple dashed lines, respectively.

## supplementary crystallographic information

### Comment

In continuation of efforts to explore the structure-activity relationships of new *S*-substituted dithiocarbazates and their metal complexes (Ravoof *et al.*, 2010; Tayamon *et al.*, 2012), the title salt (I) was obtained during an attempt to prepare the benzylhydrazine analogue of *S*-benzyldithiocarbazate.

The asymmetric unit of salt (I) comprises a 2,2-dibenzylhydrazinium cation and a chloride anion, Fig. 1. The sum of the angles about the N1 atom approximates 331° confirming its pyramidal nature. The dihedral angle between the phenyl rings is 89.95 (10)°, thereby displaying an orthogonal relationship. Hydrazinium cations are comparatively rare in the crystallographic literature with the most closely related structure being that of the diphenyl analogue, isolated as its [Au(CN)_2_^-^] salt monohydrate (Stender *et al.*, 2003). The N—N distance in this structure of 1.453 (5) Å is indistinguishable from that in (I) of 1.453 (2) Å.

The crystal packing is dominated by N—H···Cl hydrogen bonds, Table 1. Each ammonium—H atom forms a hydrogen atom with a chloride to generate an undulating ribbon along the *a* axis comprising edge-shared eight-membered {···HNH···Cl}_2_ synthons, Fig. 2. These are connected into layers in the *ab* plane by C—H···π interactions, Fig. 3 and Table 2. Layers stack along the *c* axis with no specific interactions between them.

### Experimental

The title compound was isolated as a side-product during the synthesis of a benzylhydrazine analogue of *S*-benzyldithiocarbazate using a procedure adapted from Tarafder *et al.* (2000). Potassium hydroxide (0.02 mol, 1.12 g) and benzylhydrazine (0.02 mol, 3.9 g) were each completely dissolved in chloroform (20 ml). The benzylhydrazine solution was added to the cooled mixture of potassium hydroxide. The combined solution was kept in an ice-salt bath while carbon disulfide (0.02 mol, 1.52 g) was added with constant stirring over one hour. Benzylchloride (0.02 mol, 2.3 ml) was added drop-wise to the above mixture with vigorous stirring. The initial precipitate was removed by filtration and then diethyl ether was added to the solution. A precipitate (0.63 g) was filtered from the solution after one day. Pale-yellow crystals of the title salt (*M*.pt > 583 K) were harvested from the filtrate on the second day (0.30 g).

### Refinement

Carbon-bound H-atoms were placed in calculated positions (C—H 0.95 to 0.99 Å) and were included in the refinement in the riding model approximation, with *U*_iso_(H) = 1.2*U*_equiv_(C). The nitrogen-bound H-atoms were refined freely.

### Crystal data

**Table:**

C_14_H_17_N_2_^+^·Cl^−^	*Z* = 2
*M**_r_* = 248.75	*F*(000) = 264
Triclinic, *P*1	*D*_x_ = 1.308 Mg m^−^^3^
Hall symbol: -P 1	Cu *K*α radiation, λ = 1.54180 Å
*a* = 5.6155 (4) Å	Cell parameters from 3230 reflections
*b* = 9.9804 (7) Å	θ = 4–71°
*c* = 11.7302 (9) Å	µ = 2.49 mm^−^^1^
α = 79.532 (6)°	*T* = 100 K
β = 78.508 (6)°	Plate, colourless
γ = 83.550 (6)°	0.14 × 0.09 × 0.02 mm
*V* = 631.54 (8) Å^3^	

### Data collection

**Table:**

Oxford Diffraction Xcalibur Eos Gemini diffractometer	2407 independent reflections
Radiation source: fine-focus sealed tube	2076 reflections with *I* > 2σ(*I*)
Graphite monochromator	*R*_int_ = 0.034
Detector resolution: 16.1952 pixels mm^-1^	θ_max_ = 71.5°, θ_min_ = 3.9°
ω scans	*h* = −6→6
Absorption correction: multi-scan (*CrysAlis PRO*; Agilent, 2011)	*k* = −12→11
*T*_min_ = 0.72, *T*_max_ = 0.95	*l* = −14→14
6961 measured reflections	

### Refinement

**Table:**

Refinement on *F*^2^	Primary atom site location: structure-invariant direct methods
Least-squares matrix: full	Secondary atom site location: difference Fourier map
*R*[*F*^2^ > 2σ(*F*^2^)] = 0.047	Hydrogen site location: inferred from neighbouring sites
*wR*(*F*^2^) = 0.129	H atoms treated by a mixture of independent and constrained refinement
*S* = 1.07	*w* = 1/[σ^2^(*F*_o_^2^) + (0.079*P*)^2^ + 0.1383*P*] where *P* = (*F*_o_^2^ + 2*F*_c_^2^)/3
2407 reflections	(Δ/σ)_max_ < 0.001
166 parameters	Δρ_max_ = 0.45 e Å^−^^3^
0 restraints	Δρ_min_ = −0.24 e Å^−^^3^

### Special details

**Table:**

Geometry. All s.u.'s (except the s.u. in the dihedral angle between two l.s. planes) are estimated using the full covariance matrix. The cell s.u.'s are taken into account individually in the estimation of s.u.'s in distances, angles and torsion angles; correlations between s.u.'s in cell parameters are only used when they are defined by crystal symmetry. An approximate (isotropic) treatment of cell s.u.'s is used for estimating s.u.'s involving l.s. planes.
Refinement. Refinement of *F*^2^ against ALL reflections. The weighted *R*-factor *wR* and goodness of fit *S* are based on *F*^2^, conventional *R*-factors *R* are based on *F*, with *F* set to zero for negative *F*^2^. The threshold expression of *F*^2^ > 2σ(*F*^2^) is used only for calculating *R*-factors(gt) *etc*. and is not relevant to the choice of reflections for refinement. *R*-factors based on *F*^2^ are statistically about twice as large as those based on *F*, and *R*- factors based on ALL data will be even larger.

### Fractional atomic coordinates and isotropic or equivalent isotropic displacement parameters (Å^2^)

**Table:**

	*x*	*y*	*z*	*U*_iso_*/*U*_eq_	
Cl1	0.84514 (8)	0.01710 (5)	0.33994 (4)	0.02664 (19)	
N1	0.2435 (3)	0.28342 (17)	0.37150 (14)	0.0250 (4)	
N2	0.2870 (3)	0.13600 (18)	0.40100 (16)	0.0248 (4)	
H1n	0.444 (5)	0.104 (3)	0.367 (2)	0.033 (6)*	
H2n	0.264 (4)	0.109 (3)	0.486 (2)	0.034 (6)*	
H3n	0.167 (5)	0.095 (3)	0.379 (2)	0.041 (7)*	
C1	0.2203 (4)	0.3190 (2)	0.24543 (18)	0.0279 (5)	
H1A	0.1843	0.4191	0.2271	0.033*	
H1B	0.0782	0.2754	0.2346	0.033*	
C2	0.4393 (4)	0.2781 (2)	0.15639 (17)	0.0250 (4)	
C3	0.6219 (4)	0.3662 (2)	0.10779 (19)	0.0309 (5)	
H3	0.6066	0.4551	0.1280	0.037*	
C4	0.8266 (4)	0.3253 (2)	0.02986 (19)	0.0340 (5)	
H4	0.9500	0.3863	−0.0029	0.041*	
C5	0.8508 (4)	0.1958 (2)	0.00005 (19)	0.0313 (5)	
H5	0.9908	0.1676	−0.0529	0.038*	
C6	0.6703 (4)	0.1077 (2)	0.04771 (18)	0.0301 (5)	
H6	0.6871	0.0185	0.0279	0.036*	
C7	0.4638 (4)	0.1489 (2)	0.12462 (18)	0.0281 (5)	
H7	0.3389	0.0884	0.1556	0.034*	
C8	0.4435 (4)	0.3486 (2)	0.40018 (18)	0.0269 (5)	
H8A	0.4279	0.4481	0.3709	0.032*	
H8B	0.6021	0.3107	0.3601	0.032*	
C9	0.4366 (4)	0.3245 (2)	0.53131 (18)	0.0244 (4)	
C10	0.6359 (4)	0.2601 (2)	0.57891 (19)	0.0282 (5)	
H10	0.7779	0.2302	0.5281	0.034*	
C11	0.6301 (4)	0.2389 (2)	0.70000 (19)	0.0285 (5)	
H11	0.7673	0.1944	0.7313	0.034*	
C12	0.4247 (4)	0.2827 (2)	0.77478 (18)	0.0269 (5)	
H12	0.4207	0.2693	0.8574	0.032*	
C13	0.2239 (4)	0.3465 (2)	0.72795 (18)	0.0275 (5)	
H13	0.0817	0.3759	0.7790	0.033*	
C14	0.2303 (4)	0.3675 (2)	0.60783 (18)	0.0259 (4)	
H14	0.0925	0.4117	0.5769	0.031*	

### Atomic displacement parameters (Å^2^)

**Table:**

	*U*^11^	*U*^22^	*U*^33^	*U*^12^	*U*^13^	*U*^23^
Cl1	0.0242 (3)	0.0312 (3)	0.0251 (3)	−0.00283 (19)	−0.00452 (19)	−0.0057 (2)
N1	0.0277 (9)	0.0256 (9)	0.0217 (9)	−0.0021 (7)	−0.0038 (7)	−0.0047 (7)
N2	0.0237 (10)	0.0277 (10)	0.0233 (9)	−0.0037 (7)	−0.0040 (7)	−0.0041 (7)
C1	0.0296 (11)	0.0293 (11)	0.0236 (10)	0.0006 (9)	−0.0047 (8)	−0.0032 (8)
C2	0.0274 (11)	0.0294 (11)	0.0186 (9)	−0.0006 (8)	−0.0065 (8)	−0.0035 (8)
C3	0.0404 (13)	0.0289 (11)	0.0240 (10)	−0.0048 (9)	−0.0060 (9)	−0.0048 (9)
C4	0.0356 (12)	0.0416 (14)	0.0242 (11)	−0.0113 (10)	−0.0013 (9)	−0.0035 (9)
C5	0.0276 (11)	0.0420 (13)	0.0237 (10)	0.0012 (9)	−0.0037 (8)	−0.0072 (9)
C6	0.0356 (12)	0.0308 (12)	0.0247 (10)	0.0016 (9)	−0.0072 (9)	−0.0073 (9)
C7	0.0299 (11)	0.0313 (11)	0.0236 (10)	−0.0043 (9)	−0.0058 (8)	−0.0037 (9)
C8	0.0278 (11)	0.0293 (11)	0.0234 (10)	−0.0066 (8)	−0.0010 (8)	−0.0054 (8)
C9	0.0249 (10)	0.0247 (10)	0.0243 (10)	−0.0071 (8)	−0.0016 (8)	−0.0057 (8)
C10	0.0245 (11)	0.0316 (11)	0.0279 (11)	−0.0033 (8)	0.0013 (8)	−0.0092 (9)
C11	0.0266 (11)	0.0279 (11)	0.0320 (11)	−0.0023 (8)	−0.0076 (9)	−0.0052 (9)
C12	0.0310 (11)	0.0291 (11)	0.0215 (10)	−0.0068 (9)	−0.0045 (8)	−0.0039 (8)
C13	0.0268 (11)	0.0280 (11)	0.0264 (11)	−0.0020 (8)	−0.0001 (8)	−0.0062 (8)
C14	0.0250 (10)	0.0256 (11)	0.0268 (10)	−0.0017 (8)	−0.0047 (8)	−0.0039 (8)

### Geometric parameters (Å, º)

**Table:**

N1—N2	1.453 (2)	C6—C7	1.392 (3)
N1—C8	1.481 (3)	C6—H6	0.9500
N1—C1	1.485 (3)	C7—H7	0.9500
N2—H1n	0.94 (3)	C8—C9	1.506 (3)
N2—H2n	0.97 (3)	C8—H8A	0.9900
N2—H3n	0.93 (3)	C8—H8B	0.9900
C1—C2	1.516 (3)	C9—C10	1.391 (3)
C1—H1A	0.9900	C9—C14	1.395 (3)
C1—H1B	0.9900	C10—C11	1.392 (3)
C2—C7	1.391 (3)	C10—H10	0.9500
C2—C3	1.391 (3)	C11—C12	1.383 (3)
C3—C4	1.391 (3)	C11—H11	0.9500
C3—H3	0.9500	C12—C13	1.392 (3)
C4—C5	1.385 (3)	C12—H12	0.9500
C4—H4	0.9500	C13—C14	1.380 (3)
C5—C6	1.381 (3)	C13—H13	0.9500
C5—H5	0.9500	C14—H14	0.9500
			
N2—N1—C8	108.71 (15)	C7—C6—H6	119.8
N2—N1—C1	108.85 (15)	C2—C7—C6	120.4 (2)
C8—N1—C1	113.32 (16)	C2—C7—H7	119.8
N1—N2—H1n	112.5 (16)	C6—C7—H7	119.8
N1—N2—H2n	110.1 (14)	N1—C8—C9	110.82 (17)
H1n—N2—H2n	109 (2)	N1—C8—H8A	109.5
N1—N2—H3n	108.9 (16)	C9—C8—H8A	109.5
H1n—N2—H3n	112 (2)	N1—C8—H8B	109.5
H2n—N2—H3n	104 (2)	C9—C8—H8B	109.5
N1—C1—C2	116.42 (16)	H8A—C8—H8B	108.1
N1—C1—H1A	108.2	C10—C9—C14	118.43 (19)
C2—C1—H1A	108.2	C10—C9—C8	120.94 (18)
N1—C1—H1B	108.2	C14—C9—C8	120.63 (18)
C2—C1—H1B	108.2	C11—C10—C9	120.91 (19)
H1A—C1—H1B	107.3	C11—C10—H10	119.5
C7—C2—C3	118.77 (19)	C9—C10—H10	119.5
C7—C2—C1	119.93 (19)	C12—C11—C10	120.03 (19)
C3—C2—C1	121.27 (18)	C12—C11—H11	120.0
C4—C3—C2	120.7 (2)	C10—C11—H11	120.0
C4—C3—H3	119.7	C11—C12—C13	119.43 (19)
C2—C3—H3	119.7	C11—C12—H12	120.3
C5—C4—C3	120.1 (2)	C13—C12—H12	120.3
C5—C4—H4	119.9	C14—C13—C12	120.40 (18)
C3—C4—H4	119.9	C14—C13—H13	119.8
C6—C5—C4	119.7 (2)	C12—C13—H13	119.8
C6—C5—H5	120.2	C13—C14—C9	120.80 (19)
C4—C5—H5	120.2	C13—C14—H14	119.6
C5—C6—C7	120.4 (2)	C9—C14—H14	119.6
C5—C6—H6	119.8		
			
N2—N1—C1—C2	−60.2 (2)	N2—N1—C8—C9	−66.5 (2)
C8—N1—C1—C2	60.9 (2)	C1—N1—C8—C9	172.31 (16)
N1—C1—C2—C7	87.6 (2)	N1—C8—C9—C10	122.1 (2)
N1—C1—C2—C3	−90.4 (2)	N1—C8—C9—C14	−58.4 (3)
C7—C2—C3—C4	−0.7 (3)	C14—C9—C10—C11	0.0 (3)
C1—C2—C3—C4	177.35 (19)	C8—C9—C10—C11	179.45 (19)
C2—C3—C4—C5	−0.2 (3)	C9—C10—C11—C12	−0.3 (3)
C3—C4—C5—C6	0.2 (3)	C10—C11—C12—C13	0.7 (3)
C4—C5—C6—C7	0.5 (3)	C11—C12—C13—C14	−0.7 (3)
C3—C2—C7—C6	1.5 (3)	C12—C13—C14—C9	0.4 (3)
C1—C2—C7—C6	−176.61 (18)	C10—C9—C14—C13	0.0 (3)
C5—C6—C7—C2	−1.4 (3)	C8—C9—C14—C13	−179.51 (19)

### Hydrogen-bond geometry (Å, º)

Cg1 is the centroid of the C9–C14 phenyl ring.

**Table:**

*D*—H···*A*	*D*—H	H···*A*	*D*···*A*	*D*—H···*A*
N2—H1N···Cl1	0.94 (3)	2.30 (3)	3.2130 (18)	163 (2)
N2—H2N···Cl1^i^	0.97 (2)	2.21 (2)	3.1287 (19)	158 (2)
N2—H3N···Cl1^ii^	0.93 (3)	2.20 (3)	3.1235 (18)	172 (2)
C8—H8*A*···*Cg*1^iii^	0.99	2.64	3.542 (2)	152

Symmetry codes: (i) −*x*+1, −*y*, −*z*+1; (ii) *x*−1, *y*, *z*; (iii) −*x*+1, −*y*+1, −*z*+1.
